# Geospatial screening layers for peat swamp forest loss in the Lac Télé landscape, Congo, 2001–2024

**DOI:** 10.1016/j.dib.2026.113061

**Published:** 2026-07-07

**Authors:** Armel Landry Batchi-Bouyou, Jacques Dollon Mbama Ntabi

**Affiliations:** a Washington University in St. Louis, St. Louis, MO, USA; b Université Marien Ngouabi, Brazzaville, Congo; c Public Interest Science Center, Brazzaville, Congo

**Keywords:** Peat swamp forest, Forest loss, Congo basin, Geospatial data, Remote sensing, Hansen global forest change, Peatland mapping

## Abstract

This data article describes a curated, analysis-ready geospatial data package for screening peat swamp forest loss in the Lac Télé protected-area complex and surrounding 50 km landscape buffer in the Republic of the Congo. The package integrates a simplified CongoPeat peatland classification with Hansen Global Forest Change (GFC) v1.12 tree-cover and loss-year layers (2000–2024), and provides derived products that screen recent forest-cover loss on peat swamp forest between 2015 and 2024. The package includes machine-readable quality-control summaries, annual loss time series for 2001–2024, sensitivity analyses across forest-cover thresholds, and stratified validation points with Google Earth visual interpretation labels. These resources support reuse in protected-area monitoring, peatland conservation planning, and validation design across Central African peatland landscapes.

Specifications Table

**Table:** 

Subject	Earth & Environmental Sciences
Specific subject area	Geospatial monitoring of tropical peatland forest disturbance
Type of data	GeoTIFF raster, GeoPackage vector, CSV table, PNG image
Data collection	Secondary data derived from Hansen/UMD Global Forest Change v1.12 (2000–2024), CongoPeat simplified peatland map, WDPA protected-area boundaries, and manual visual interpretation of stratified validation points using Google Earth web imagery. Processed using R with terra, sf, exactextractr, and reproducible tabular export routines
Data source location	Lac Télé Community Reserve and 50 km buffer, Likouala Department, Republic of the Congo (approx. 1.3°N, 17.2°E)
Data accessibility	Repository name: Zenodo Data identification number: Dataset DOI: 10.5281/zenodo.20508051; Dataset concept DOI: 10.5281/zenodo.18770920; Code/software DOI: 10.5281/zenodo.18773511 Direct URL to data: https://doi.org/10.5281/zenodo.18770920 Data are publicly available for download without restriction. The revised dataset archive includes geospatial raster and vector outputs, tabular summaries, validation-point files, Google Earth visual-validation support materials, confusion matrices, accuracy metrics, area-adjusted estimates, sensitivity analyses, annual loss time-series outputs, metadata, and checksums. The associated reproducible R workflow is archived separately through the GitHub-Zenodo software record. Users who wish to reproduce the full workflow should additionally obtain the WDPA boundary for site_id 166739 from Protected Planet.
Related research article	None (direct submission)

## 1 Value of the Data

• These data provide analysis-ready, landscape-clipped rasters and tabular summaries that eliminate the need for researchers to independently download, preprocess, and harmonize large global remote-sensing tiles for the Lac Télé landscape.
• Conservation practitioners, protected-area managers, and policymakers working on Central African peatlands can use the screening layers and annual time series to rapidly assess forest-loss trends on peat swamp forest without specialized geospatial expertise.
• The labelled stratified validation points, Google Earth visual interpretation notes, and sensitivity analyses across multiple forest-cover thresholds enable researchers to assess the plausibility of mapped forest-loss signals and to design formal accuracy assessments for carbon accounting and land-use planning.
• The fully documented and reproducible R Markdown workflow allows other research groups to adapt the processing pipeline to additional protected areas or peatland regions in the Congo Basin and beyond.
• The versioned Zenodo archive with persistent DOIs supports transparent updating when new annual Global Forest Change releases become available, facilitating long-term monitoring continuity.

## 2 Background

The Lac Télé Community Reserve and surrounding Likouala landscape in the Republic of the Congo harbour some of the largest tropical peatland deposits on Earth, yet spatially explicit monitoring of forest disturbance on peat remains limited. Global remote-sensing products such as the Hansen Global Forest Change dataset provide annual forest-loss detection at 30 m resolution, and the CongoPeat mapping project has produced peatland extent layers, but these resources are distributed as large global or regional tiles that require substantial preprocessing before they can be used for site-level screening. This data article provides a curated, analysis-ready geospatial package that harmonizes these upstream products for the Lac Télé landscape, enabling rapid screening of peat swamp forest loss without the overhead of independent data acquisition and alignment. The accompanying reproducible workflow and machine-readable diagnostics support transparent reuse and updating as new data releases become available.

## 3 Data Description

The dataset is distributed as a versioned Zenodo record [1] containing a single archival package (outputs_public.zip), together with a top-level README_dataset.txt. The archive is organized around shareable outputs: (1) landscape-clipped rasters and screening masks, (2) tabular summaries and QA diagnostics, (3) stratified validation-point files with manual visual interpretation labels, and (4) figure outputs for rapid inspection and reporting. All raster layers are provided as GeoTIFFs in EPSG:4326, and validation points are provided as CSV and GeoPackage (EPSG:4326). Users can enter at multiple levels depending on their needs: users interested in reproducible headline summaries can begin with the tabular outputs; users interested in GIS-based exploration can work directly from the clipped rasters; users interested in validation design can start from the stratified point sample.

### 3.1 Raster products

**Fig. 1:**
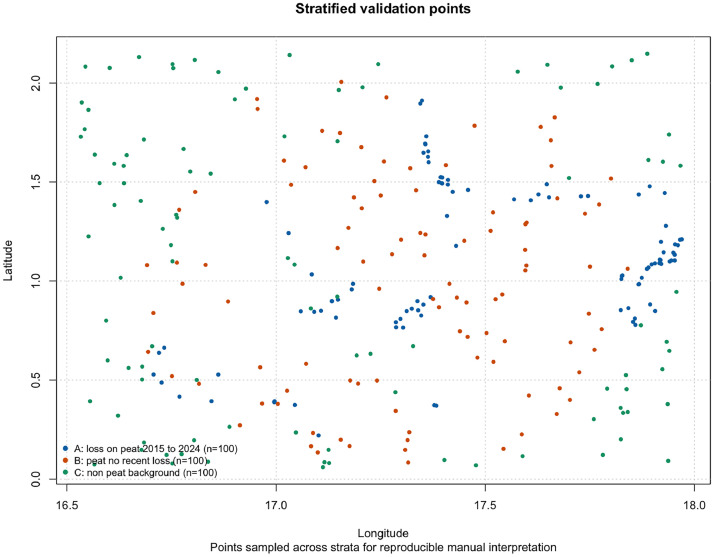
Study area and spatial distribution of the stratified validation points in the Lac Télé landscape. The sample contains 300 points, with 100 points in each of three strata: mapped recent loss on peat swamp forest during 2015–2024, peat swamp forest with no recent mapped loss, and non-peat background.

**Fig. 2:**
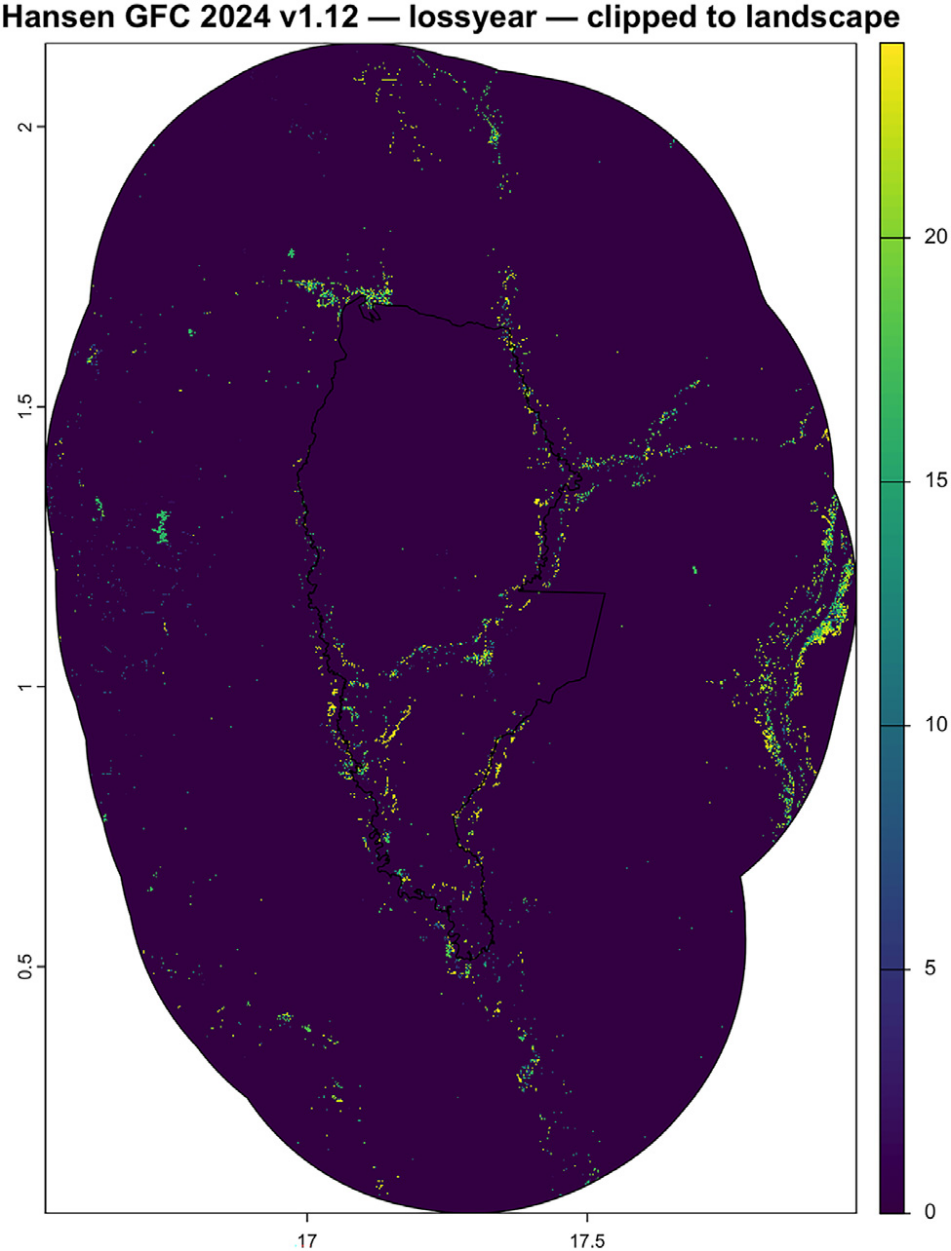
Screening mask of mapped forest-cover loss during 2015–2024 overlapping peat swamp forest in the Lac Télé landscape. The image corresponds to the binary raster loss_on_peat_swamp_2015_2024_clip_landscape.tif.

peat_100m_clip_landscape.tif is the CongoPeat simplified map clipped to the 50 km landscape buffer, retaining the original categorical class encoding (1–4 plus NoData). treecover2000_clip_landscape.tif is the Hansen tree-cover layer clipped to the same landscape extent, preserving the original 0–100 canopy-cover encoding. lossyear_clip_landscape.tif is the Hansen lossyear layer clipped to the landscape buffer, preserving the original integer coding (0–24). loss_on_peat_swamp_2015_2024_clip_landscape.tif is the key derived screening product: a binary indicator raster (0/1) identifying pixels that simultaneously satisfy peat swamp forest (CongoPeat class 4) and mapped forest loss during 2015–2024. Each raster product has a corresponding quick-look PNG. Quick-look maps (Fig. 1, Fig. 2) provide qualitative confirmation that rasters are clipped to the same landscape buffer extent and that spatial patterns are plausible.

### 3.2 Tabular summaries and diagnostics

summary_metrics_corrected.csv is a one-row configuration and metrics record capturing key workflow parameters and corresponding map-based screening metrics in hectares. annual_loss_timeseries_2001_2024.csv provides annual mapped loss summaries in wide format; a tidy long version is provided as annual_loss_timeseries_2001_2024_LONG.csv. manual_visual_validation.csv provides the labelled visual interpretation table for the stratified validation points. This file records the model stratum, point identifier, geographic coordinates, reference class assigned from Google Earth web imagery, imagery source, and a short visual interpretation note for each point. qa_raster_summary.csv provides quality-assurance diagnostics (Fig. S3). peat_class_frequency.csv (Fig. S4) and lossyear_value_frequency.csv (Fig. S5) provide categorical frequency tables.

### 3.3 Validation points

validation_points_stratified.csv and validation_points_stratified.gpkg provide the reproducible stratified point sample used for visual validation. The revised release contains 300 points, with 100 points in each of three strata: (A) mapped recent loss on peat swamp forest during 2015–2024, (B) peat swamp forest with no recent mapped loss, and (C) non-peat background. The labelled file manual_visual_validation.csv adds the Google Earth web imagery interpretation for each point, including reference class, imagery source, and a short interpretation note. The spatial distribution of the validation points is shown in Fig. 1.

## 4 Experimental Design, Materials and Methods

All data products were generated using a single, corruption-tolerant R Markdown workflow (WWL_V7_compliance.Rmd) that implements deterministic inputs, explicit file paths, and auditable intermediate outputs. The workflow was designed to (i) ingest a protected-area boundary (WDPA site_id = 166739, locally provided), (ii) download and integrity-check upstream raster tiles, (iii) harmonize coordinate reference systems and pixel grids, (iv) clip source layers to a consistent landscape extent, (v) compute derived screening masks and map-based summaries, and (vi) export tabular diagnostics, figures, stratified validation-sample points, and manual visual interpretation labels. Shareable outputs are bundled into a single public archive (outputs_public.zip) that excludes restricted inputs such as WDPA boundary geometry.

The workflow was executed in R on a local workstation using widely adopted open-source geospatial packages. Core spatial operations used terra (raster I/O, reprojection, masking, cell-area computation), sf (vector I/O and buffering), and exactextractr/terra-based summarization routines for area aggregation. Tabular manipulation used dplyr/tidyr and readr, while ggplot2 provided figure generation.

### 4.1 Study area definition and boundary derivation

The protected-area boundary was obtained from the World Database on Protected Areas (WDPA) [2] for site_id 166739. Because WDPA terms prohibit redistributing WDPA geometry via downloadable archives, the downloadable dataset does not include WDPA boundary geometry or the derived 50 km buffer polygon. Users should obtain the boundary via ProtectedPlanet.net and rerun the workflow to regenerate the boundary and buffer locally. All raster outputs are aligned to EPSG:4326 (WGS 84), matching the native coordinate reference system of the Hansen GFC products. The study area boundary and landscape buffer are shown in Fig. 1.

### 4.2 Input raster datasets

Annual forest-loss screening used the Hansen/UMD Global Forest Change (GFC) dataset (v1.12, 2000–2024) [3,4]. Two layers were used: (i) tree canopy cover in 2000 (treecover2000; encoded 0–100 as percent canopy closure for vegetation taller than 5 m) and (ii) lossyear (encoded as 0 for no loss and 1–24 for loss detected primarily in 2001–2024). Source tiles are downloaded by the accompanying workflow as needed. To minimize archive size, the downloadable deposit contains only the landscape-clipped rasters and derived screening layers plus machine-readable summaries; the workflow can re-download upstream tiles to reproduce or update outputs.

Peat swamp forest screening used CongoPeat map products [5]. The default workflow uses the simplified categorical peat/land-cover map (four-class encoding: 1 = open water, 2 = savanna, 3 = other tropical forest/terra firme, 4 = peat swamp forest) at 100 m resolution and treats class 4 as a binary peat mask. CongoPeat metadata indicates that simplified peat maps are intended for display and rough calculations; for more precise area/overlap calculations, users should use the combined classification-and-probability product and apply an explicit peat-probability threshold. The workflow supports both options and records which option was used.

### 4.3 Coordinate system harmonization and grid alignment

All spatial layers were processed in EPSG:4326. Because the Hansen GFC rasters are delivered in a geographic grid (1 arc-second per pixel; approximately 30 m at the equator) and the CongoPeat simplified map is delivered at 100 m resolution, direct overlay requires careful handling of differing resolutions and pixel boundaries. The workflow applies a two-stage alignment strategy.

First, each source raster was cropped and masked to the landscape buffer at its native resolution, producing peat_100m_clip_landscape.tif (100 m), lossyear_clip_landscape.tif (≈30 m), and treecover2000_clip_landscape.tif (≈30 m). Quick-look map images were exported in parallel to support visual inspection.

Second, for overlay computation, the peat map (class 4) was resampled to the Hansen grid using nearest-neighbour resampling, ensuring that the categorical peat classes are preserved without artificial mixing. The resampled peat mask was then combined with lossyear to identify loss pixels occurring on peat swamp forest.

### 4.4 Forest baseline definition and sensitivity thresholds

Baseline forest extent was defined using the Hansen treecover2000 layer and a canopy-cover threshold of 30%. Pixels with treecover2000 ≥ 30 were treated as baseline forest in 2000. Because forest-definition thresholds can materially affect area-based summaries, the workflow additionally computes a sensitivity analysis across three commonly used thresholds (10%, 30%, and 50% canopy cover). Sensitivity results are exported as sensitivity_forest_threshold_10_30_50.csv (Fig. S1).

### 4.5 Derivation of loss products and temporal summaries

The lossyear source tile was clipped to the landscape buffer to produce lossyear_clip_landscape.tif. Values were retained as 0–24, where 0 indicates no mapped stand-replacement loss and 1–24 map to years 2001–2024 [[3], [4], [5]].

The key derived screening layer is loss_on_peat_swamp_2015_2024_clip_landscape.tif, a binary (0/1) raster at the Hansen grid resolution indicating pixels that satisfy two conditions: (i) peat swamp forest (class 4) in the CongoPeat simplified map and (ii) mapped forest loss in the lossyear layer during 2015–2024 (lossyear values 15–24). The spatial distribution of this screening layer is shown in Fig. 2.

Because rasters are stored in geographic coordinates, pixel area varies with latitude. The workflow computes per-cell areas using geodesic cell-area estimates and aggregates mapped areas in hectares. These mapped areas are intended as screening metrics; consistent with Hansen GFC guidance, they should not be treated as definitive area estimates without a validation-based accuracy/area estimation framework.

**Fig. 3:**
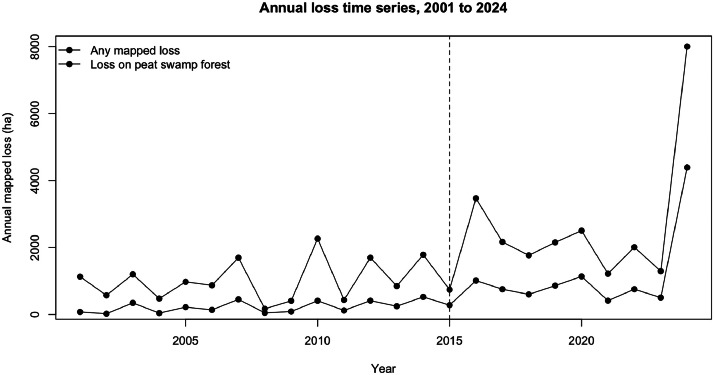
Annual mapped forest-loss time series from 2001 to 2024 for peat swamp forest and baseline forest within the landscape buffer and protected-area boundary.

Annual loss time series were generated for (a) loss on peat swamp forest and (b) loss on baseline forest, each summarized for two spatial subsets: the landscape buffer and the protected-area boundary (Fig. 3). The wide-format time series is exported as annual_loss_timeseries_2001_2024.csv. A long/tidy version is exported as annual_loss_timeseries_2001_2024_LONG.csv.

A one-row canonical metrics table is exported as summary_metrics_corrected.csv. This file records the site identifier, buffer distance, source tile label, peat class used, baseline forest threshold, area estimates for peat swamp forest and baseline forest, total loss across 2001–2024, and the subset of loss occurring in 2015–2024 for both landscape and protected-area subsets.

### 4.6 Quality assurance outputs

Several machine-readable diagnostics were generated to allow users to verify that rasters were read correctly, have expected value ranges, and have plausible missingness. A compact raster QA table is exported as qa_raster_summary.csv (Fig. S3), which includes the number of cells (ncell), missingness fraction (na_fraction), and minimum/maximum values for key layers. Two frequency tables were also exported: peat class distribution (peat_class_frequency.csv; Fig. S4) and distribution of lossyear values (lossyear_value_frequency.csv; Fig. S5).

Automated range and missingness checks confirm that the clipped peat layer shows categorical values bounded between 1 and 4, the clipped Hansen layers show value ranges consistent with their encodings (lossyear min 0, max 24; treecover2000 min 0, max 100), and the binary derived mask shows min 0, max 1. The peat class frequency table reports non-zero counts for all four simplified classes, with peat swamp forest (class 4) dominating within the clipped landscape. The lossyear frequency table confirms that the full encoding range 1–24 is present and that 98.6% of cells carry no mapped loss.

### 4.7 Stratified validation-point sample

A reproducible stratified set of validation points was generated to support manual visual interpretation of the mapped forest-loss screening layer. The workflow defines three strata: (A) recent loss on peat swamp forest during 2015–2024, (B) peat swamp forest with no recent mapped loss, and (C) non-peat background. Points were sampled at raster-cell centres and exported as a tabular file (validation_points_stratified.csv) and as a spatial GeoPackage (validation_points_stratified.gpkg). The revised release contains 300 validation points, with 100 points per stratum. The spatial distribution of validation points is shown in Fig. 1.

### 4.8 Manual visual interpretation using google earth imagery

Each stratified validation point was manually inspected using Google Earth web imagery. For each point, we recorded a reference class, the imagery source, and a short interpretation note. Points were coded as reference_loss_on_peat when visible canopy removal, large clearings, burned areas, or recent canopy disturbance were visible around the sampled location. Points were coded as reference_not_loss_on_peat when intact forest canopy, flooded forest, or wetland vegetation was visible without clear evidence of recent clearing. Points with cloudy, low-resolution, or otherwise ambiguous imagery were flagged as skip_uncertain. The manually labelled table is provided as manual_visual_validation.csv. These labels are intended as a screening-level visual validation resource and not as a final design-based area-adjustment estimate.

### 4.9 Download integrity checks and reproducibility safeguards

Large GeoTIFF downloads can occasionally fail silently. To prevent propagation of corrupted inputs into downstream products, the workflow implements two safeguards: (i) it checks for expected existence and non-trivial file size of each downloaded tile and fails fast if size is implausibly small, and (ii) it attempts to open/read the raster using terra routines; if the raster cannot be opened, the script deletes the local file and re-downloads the tile.

### 4.10 Handling NoData and masking conventions

The Hansen GeoTIFF tiles are distributed with unsigned 8-bit encodings (0–100 for treecover2000; 0–24 for lossyear). The CongoPeat simplified peat map uses categorical integer encodings. During clipping, the workflow applies explicit masks derived from the landscape buffer geometry to all rasters, producing consistent spatial extents while retaining original value encodings.

### 4.11 Temporal windowing and event-year selection

The workflow distinguishes between any mapped loss during 2001–2024 and recent loss during 2015–2024, motivated by the operational needs of protected-area screening and near-term management planning. All summary tables include both long-run and recent-decade aggregates.

### 4.12 Internal consistency checks

Total mapped loss (2001–2024) in the landscape buffer is 39,850 ha, and recent loss (2015–2024) is a strict subset (25,324 ha). The same subset relationship holds within the protected area (7833 ha vs. 4954 ha). Loss on peat swamp forest is bounded above by total loss by construction; the reported loss on peat in the landscape for 2015–2024 (10,705 ha) is less than total loss in the same window (25,324 ha). These checks support the correctness of joins between lossyear, forest baseline masks, peat masks, and boundary subsets.

### 4.13 Visual-validation consistency

Manual interpretation of the stratified validation points provided an additional qualitative check on the mapped loss signal. The Google Earth labels showed that visually assessable points classified as recent loss on peat corresponded to visible canopy disturbance, clearing, burned areas, or related land-cover change around the sampled locations. Points assigned to the no-recent-loss peat stratum generally showed intact forest canopy, flooded forest, or wetland vegetation without clear evidence of recent clearing. These results support the plausibility of the screening layer while remaining distinct from a formal design-based accuracy assessment.

### 4.14 Temporal consistency

The annual time-series table includes 24 rows (years 2001–2024), and the tidy long version includes 96 rows (24 years × 4 metrics). The presence of a complete year sequence confirms correct decoding of lossyear values (Fig. 3; Fig. S2).

### 4.15 Sensitivity analysis for baseline forest definition

Forest area and mapped loss summaries depend on the canopy-cover threshold used to define baseline forest. Sensitivity results at 10%, 30%, and 50% thresholds (Fig. S1) show a stable pattern: landscape recent-loss fraction remains below 1% across thresholds (approximately 0.89–0.94%) and the protected-area fraction remains slightly above 1% (approximately 1.10–1.24%). This demonstrates that headline comparisons are not artefacts of a single arbitrary threshold.

### 4.16 Resolution and CRS checks

Automated metadata extraction confirms that all GeoTIFF outputs retain EPSG:4326 and expected pixel sizes. The Hansen source tiles have a 0.00025° × 0.00025° grid (≈1 arc-second), while the CongoPeat simplified map uses a coarser ≈0.000889° grid (≈100 m).

## Limitations

Users should interpret derived loss products as screening layers rather than definitive deforestation estimates. The Hansen lossyear layer represents stand-replacement disturbance [[3], [4], [5]] and is known to include both anthropogenic clearing and natural disturbances; it may miss low-intensity degradation. The GFC v1.12 update incorporates methodological changes and reprocessing from 2011 onward [4]. The CongoPeat simplified map is a categorical abstraction [5] and may not capture fine-scale peat heterogeneity. WDPA boundary geometry cannot be redistributed, so users must obtain it independently to reproduce the full workflow. For applications requiring definitive area estimates, the Google Earth visual interpretation should be treated as a screening-level validation step. Further work should extend it by applying a formal design-based accuracy and area-estimation framework, computing confusion matrices and adjusted area estimates using standard accuracy-assessment methods [6,7], and comparing screened loss patterns with independent datasets or field observations where available.

## Ethics Statement

This work uses publicly available remote-sensing and protected-area datasets. No human subjects or animal research was involved. All upstream data sources are used in compliance with their respective license terms. The authors have read and follow the ethical requirements for publication in Data in Brief and confirm that the current work does not involve human subjects, animal experiments, or any data collected from social media platforms.

## Credit Author Statement

**Armel Landry Batchi-Bouyou:** Conceptualization, Methodology, Software, Data curation, Formal analysis, Writing – original draft, Visualization. **Jacques Dollon Mbama Ntabi:** Supervision, Writing – review & editing.

## Data Availability

ZenodoGeospatial raster, vector and stratified validation-point data for peat swamp forest and forest-cover loss screening in the Lac Télé-Likouala-aux-Herbes landscape, Republic of the Congo, 2001–2024 (Original data).

## Appendix Supplementary materials

Image, application 1
