# Supplementary material for: Geospatial screening layers for peat swamp forest loss in the Lac Télé landscape, Congo, 2001–2024
Source: Data Brief. 2026 Jul 7;67:113061. doi: 10.1016/j.dib.2026.113061 (PMC13382606; doi:10.1016/j.dib.2026.113061)

**Supplementary Figure S1.** Sensitivity of baseline-forest area and recent-loss fraction to alternative treecover2000 thresholds of 10%, 30%, and 50%.

**Supplementary Figure S2.** Annual mapped forest-loss time series from 2001 to 2024, showing the landscape-buffer and protected-area subsets.

**Supplementary Figure S3.** Raster quality-assurance summary showing missing-data fractions and observed value ranges for the clipped source layers and derived binary overlay.

**Supplementary Figure S4.** Frequency distribution of the CongoPeat simplified land-cover classes within the clipped Lac Télé landscape. Classes are encoded as 1, open water; 2, savanna; 3, other tropical forest or terra firme; and 4, peat swamp forest.

**Supplementary Figure S5.** Frequency distribution of Hansen Global Forest Change loss-year values within the clipped Lac Télé landscape. A value of 0 indicates no mapped loss, while values 1–24 correspond to mapped loss during 2001–2024.


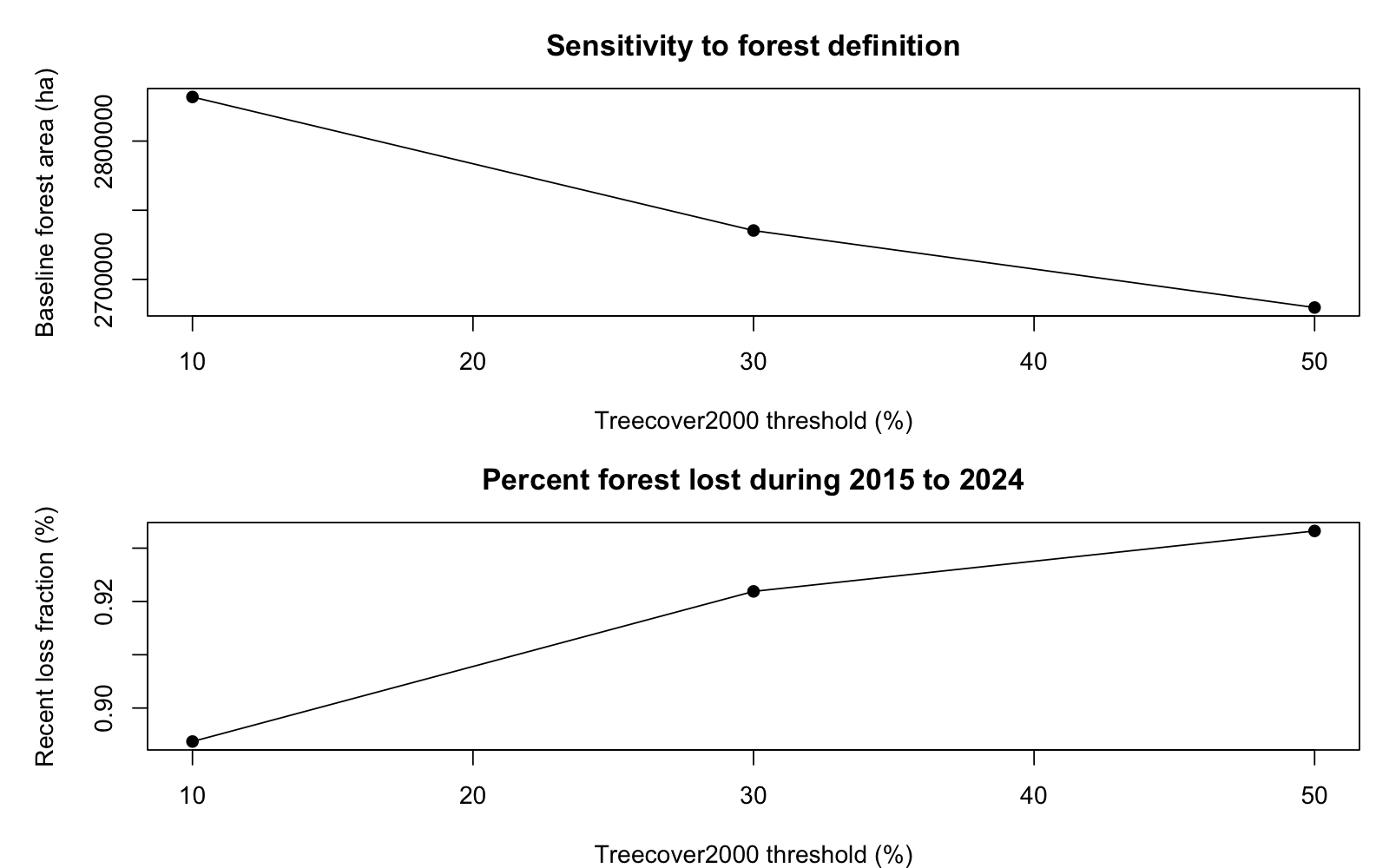


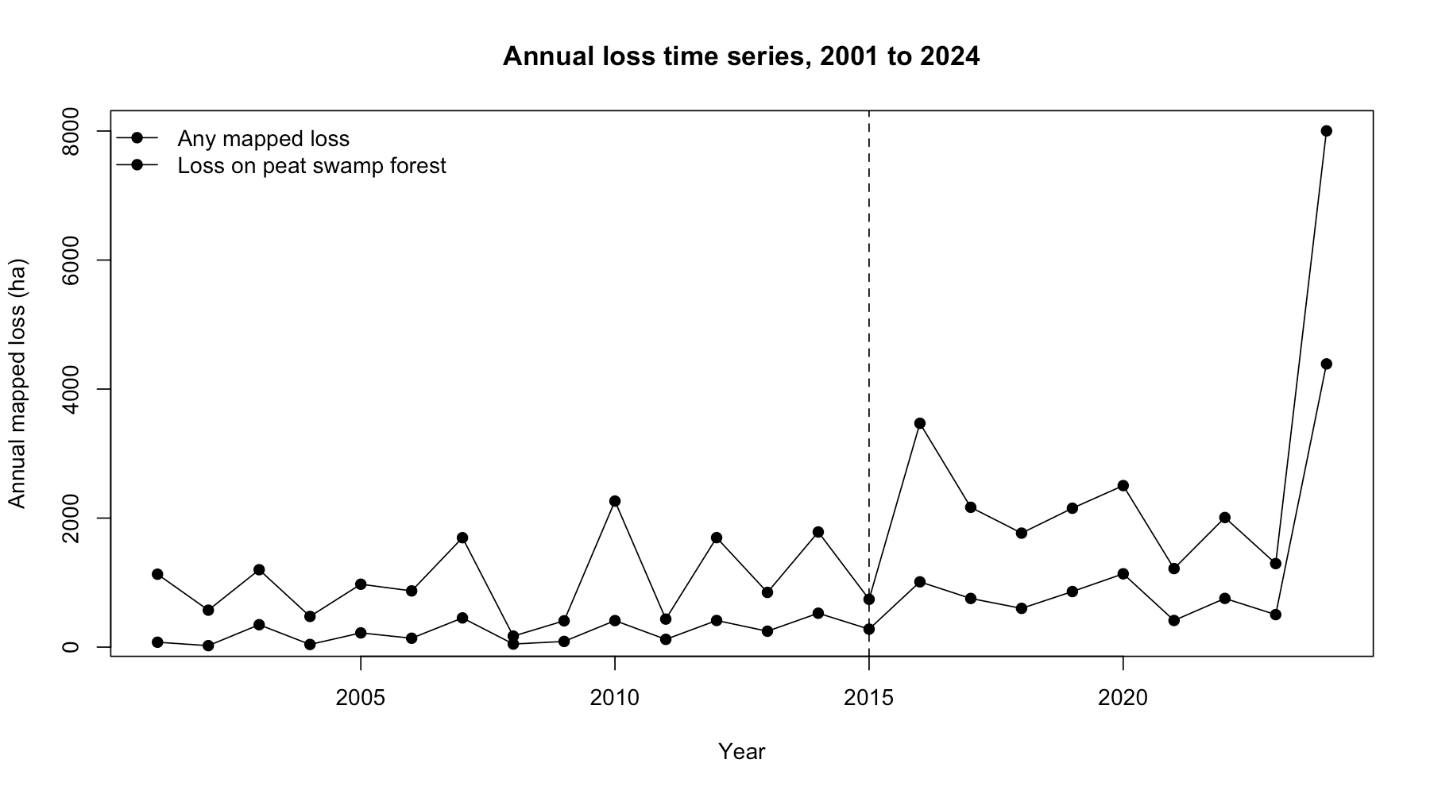


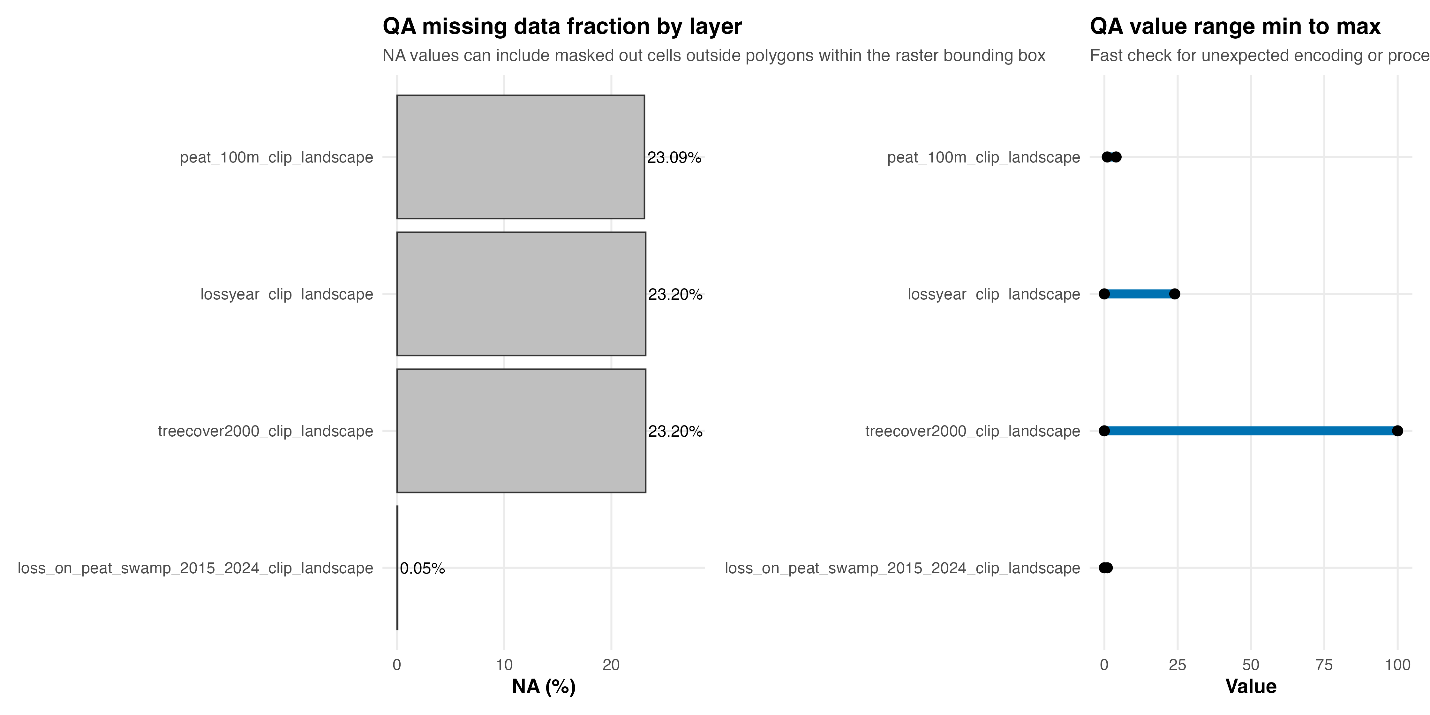


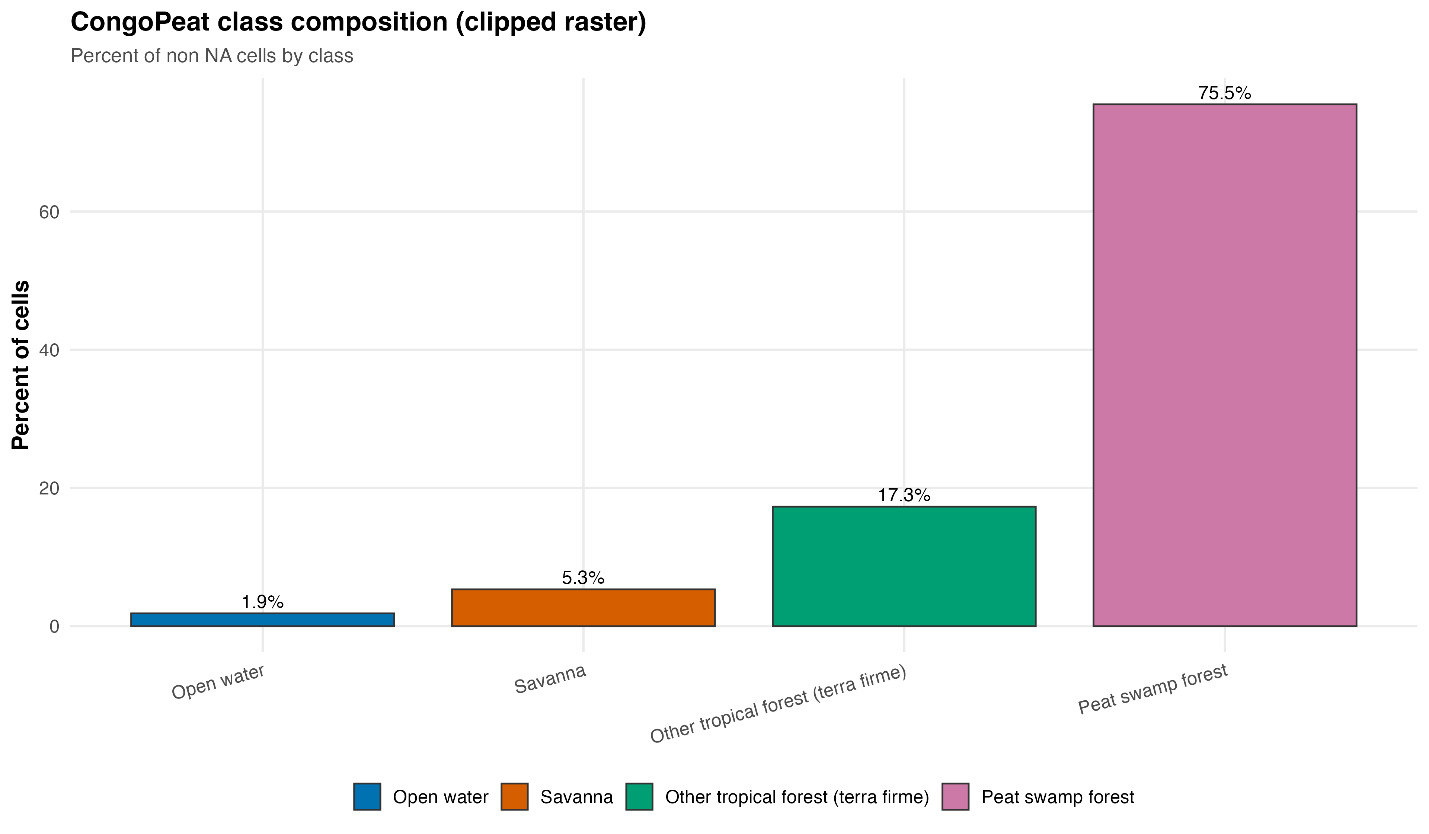


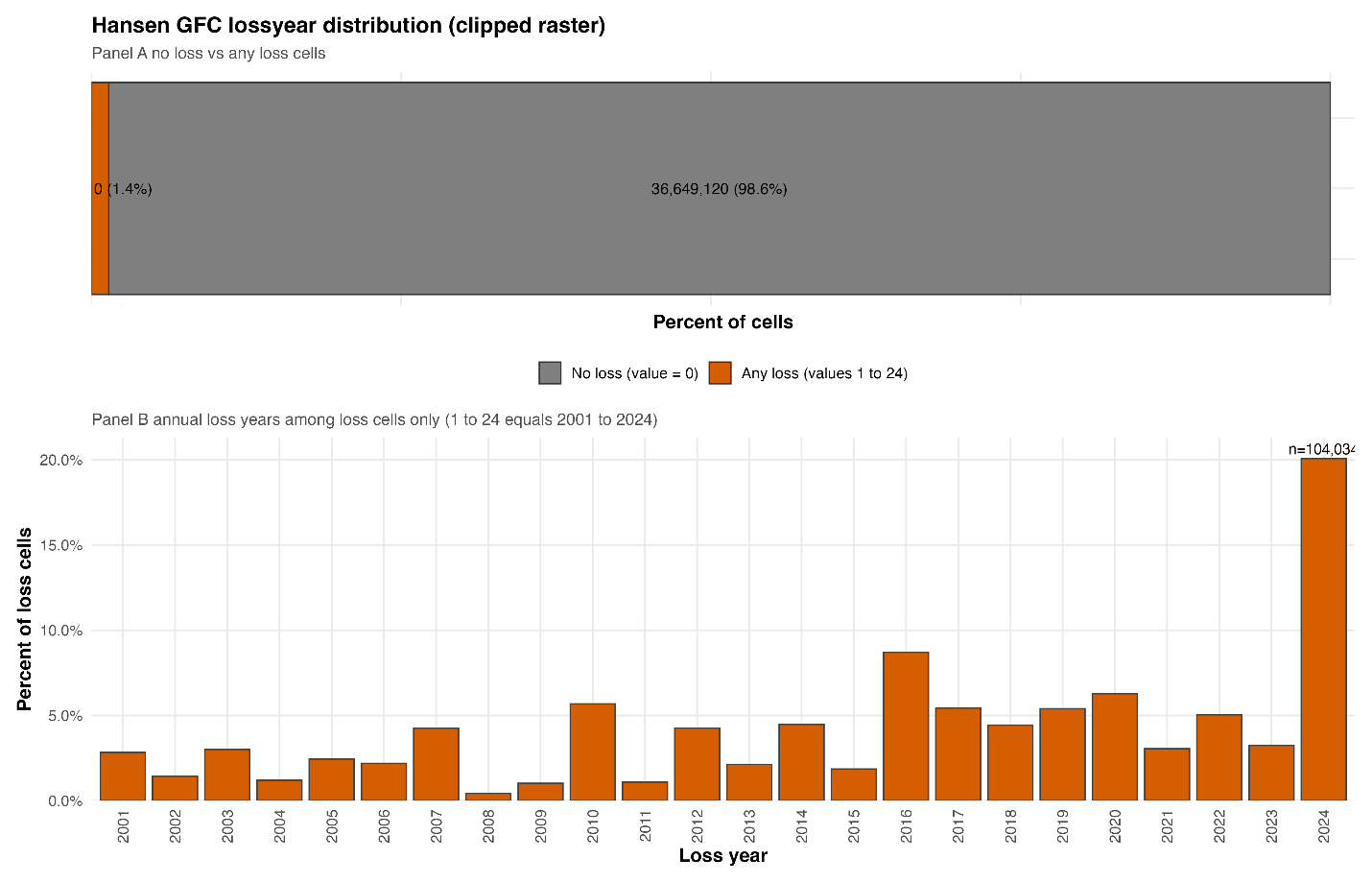

Supplement: Supplementary file 1 [file mmc1.docx]
